# Beyond Contractures in Spinal Muscular Atrophy: Identifying Lower-Limb Joint Hypermobility

**DOI:** 10.3390/jcm13092634

**Published:** 2024-04-30

**Authors:** Elizabeth R. Harding, Cara H. Kanner, Amy Pasternak, Allan M. Glanzman, Sally Dunaway Young, Ashwini K. Rao, Michael P. McDermott, Zarazuela Zolkipli-Cunningham, John W. Day, Richard S. Finkel, Basil T. Darras, Darryl C. De Vivo, Jacqueline Montes

**Affiliations:** 1 Department of Rehabilitation and Regenerative Medicine, Columbia University Irving Medical Center, New York, NY 10032, USAakr7@cumc.columbia.edu (A.K.R.); jm598@cumc.columbia.edu (J.M.); 2 Department of Neurology, Boston Children’s Hospital, Harvard Medical School, Boston, MA 02115, USA; amy.pasternak@childrens.harvard.edu (A.P.); basil.darras@childrens.harvard.edu (B.T.D.); 3 Department of Physical and Occupational Therapy Services, Boston Children’s Hospital, Boston, MA 02115, USA; 4 Department of Physical Therapy, Children’s Hospital of Philadelphia, Philadelphia, PA 19104, USA; glanzmana@chop.edu; 5 Department of Neurology and Clinical Neurosciences, Stanford University, Palo Alto, CA 94304, USA; sdy@stanford.edu (S.D.Y.); jwday@stanford.edu (J.W.D.); 6 Department of Biostatistics and Computational Biology, University of Rochester, Rochester, NY 14642, USA; michael_mcdermott@urmc.rochester.edu; 7 Division of Human Genetics, The Children’s Hospital of Philadelphia, Philadelphia, PA 19104, USA; zolkipliz@chop.edu; 8 Center for Experimental Neurotherapeutics, Department of Pediatric Medicine, St. Jude Children’s Research Hospital, Memphis, TN 38105, USA; richard.finkel@stjude.org; 9 Department of Neurology, Columbia University Irving Medical Center, New York, NY 10032, USA; dcd1@cumc.columbia.edu

**Keywords:** spinal muscular atrophy, range of motion, joint, hypermobility, contractures, function, rehabilitation

## Abstract

**Background:** The natural history of spinal muscular atrophy (SMA) is well understood, with progressive muscle weakness resulting in declines in function. The development of contractures is common and negatively impacts function. Clinically, joint hypermobility (JH) is observed but is poorly described, and its relationship with function is unknown. **Methods***:* Lower-limb ROM (range of motion) assessments of extension and flexion at the hip, knee, and ankle were performed. ROMs exceeding the published norms were included in the analysis. The functional assessments performed included the six-minute walk test (6 MWT) and the Hammersmith Functional Motor Scale—Expanded (HFMSE). **Results:** Of the 143 participants, 86% (*n* = 123) had at least one ROM measure that was hypermobile, and 22% (*n* = 32) had three or more. The HFMSE scores were inversely correlated with hip extension JH (r = −0.60, *p* = 0.21; *n* = 6) and positively correlated with knee flexion JH (r = 0.24, *p* = 0.02, *n* = 89). There was a moderate, inverse relationship between the 6 MWT distance and ankle plantar flexion JH (r = −0.73, *p* = 0.002; *n* = 15). **Conclusions:** JH was identified in nearly all participants in at least one joint in this study. Hip extension, knee flexion and ankle plantar flexion JH was associated with function. A further understanding of the trajectory of lower-limb joint ROM is needed to improve future rehabilitation strategies.

## 1. Introduction

Spinal muscular atrophy (SMA) is a rare, autosomal recessive genetic disease caused by a deficiency of the motor neuron protein called survival motor neuron (SMN). SMN is ubiquitously expressed in the human body, and a deficiency of this protein has wide-ranging effects on individuals with SMA; however, the anterior horn cells are known to be the most vulnerable [1]. The effects are progressive in nature and known to degenerate over time, leading to downstream effects on the motor units. The motor units in SMA undergo significant changes compared to healthy individuals, with the depletion of SMN also affecting neuromuscular junction maturation, protein homeostasis, muscle fiber size and function and joint tissue [2,3,4,5]. Symptoms of SMA may develop in infancy, and due to the progressive nature of SMA, untreated individuals present with notable proximal muscle weakness, delayed development, fatigue and declines in function [6]. The clinical classification of SMA (type I, type II, and type III) is determined based on the maximal functional status achieved by individuals and the age at symptom onset [7,8]. Type I is the most severe, with an early onset and individuals being unable to sit unsupported. Type II is the intermediate type, with individuals having achieved independent sitting, and type III is the mild type, with individuals having achieved independent walking [7,8]. The copy number of the SMN2 gene, a homologous copy of the SMN1 gene, is considered to be the most important factor in determining the phenotype in SMA [9]. There is an inverse relationship between the SMN2 copy number and disease severity in SMA, with type I often having two copies of SMN2, type II having three copies and type III having three or four [9]. Until recently, treatments for SMA were not available. The development of disease modifying therapies (DMTs) and increased access to them have led to a shift in treatment paradigms.

Limited joint range of motion (ROM) and the development of contractures are common and associated with weakness, immobility and diminished motor ability in individuals with SMA [10,11,12]. In a cohort of people with type II SMA, lower-limb contractures developed early and increased progressively with age [11]. Conventionally, preventive and restorative treatment for ROM has involved positioning, orthoses, stretching and ROM exercises [13]. Concurrently, joint hypermobility (JH) is observed in individuals with SMA, although it is not well described and its relationship to function is unknown [14].

Definitions of JH have lacked consensus, which has previously limited research [15,16,17]. However, efforts have been made to define the terms and differentiate aspects of JH, which has enabled the characterization of joint ROM and comparison between different groups [15,17]. Castori et al. developed a framework where JH was determined to occur in a single joint and is different from generalized joint hypermobility (GJH), which is defined as JH in all four limbs plus the axial skeleton [15,18]. Further, laxity, or hyperlaxity, is understood to describe general soft tissue characteristics rather than the functional or dynamic characteristics of the joint. Hypermobility indicates that a joint can be moved passively beyond what is defined as the normal range [17,19]. While JH is often evaluated with goniometry, the Beighton score is a widely accepted measure of GJH, with different cutoff scores for children and adults [18,19]. There is a lack of consensus on the measures that define normal and hypermobile ROM [19,20,21,22,23], as well as whether it is necessary to adjust for age, sex, and race [24]. However, reliable and expansive normative reference values have been established to enable such comparisons, specifically in neuromuscular diseases [24].

In SMA, the patterns of limitations in lower-limb ROM differ across functional levels, with the knees being most affected in type II and the ankle most affected in type III SMA [12]. Prospective studies evaluating lower-limb ROM have shown that individuals with SMA who are ambulant (type III) rarely develop hip and knee flexion contractures; however, most who are non-ambulant (type II) do develop hip and knee contractures, and they often evolve rapidly following the loss of ambulation in type III patients [25,26]. 

The prevalence of JH in the lower extremities in SMA and its relationship to function have not been described. The purpose of this study is to characterize JH in the lower limbs at the hip, knee, and ankle of the lower limbs in a large cohort of untreated children and adults with SMA and evaluate its relationship to gross motor and ambulatory function.

## 2. Methods

### 2.1. Setting

This cross-sectional study included an analysis of existing natural history data that were collected across 4 sites in the USA that form the Pediatric Neuromuscular Clinical Research Network for SMA. Data collection was approved by the Institutional Review Board or ethics committee at each location (Columbia, AAAE8252, 11/06/2009; Boston, 05-02-028, 1/25/2011; CHOP, IRB 05-00432, 8/09/2005; Stanford, IRB-31140, 9/23/2014), and consent or assent was provided by participants or their guardians, as appropriate. Range of motion and gross motor function assessments were collected by trained physical therapists who completed study-specific in-person and remote training. A manual of procedures was created and provided to participating physical therapists based on standard goniometric measurements, which is a reliable method of measuring ROM in neuromuscular disorders [10,27,28,29]. The manual included typical goniometric measurement procedures as well as suggested modifications or strategies to standardize the measurements in this heterogeneous population where typical protocols are not always feasible due to positioning challenges from contractures.

### 2.2. Participants

Participants of any age were included in the initial natural history study, provided they had genetic confirmation of SMA before 19 years of age and their SMA type was determined clinically prior to assessment. Participants were excluded if they had medical conditions that would preclude participation, a significant respiratory condition that would interfere with safe travel to the site of evaluation, and the patient’s location being beyond a reasonable driving distance, as determined by the site investigator. 

### 2.3. Assessments 

The measures were obtained during clinical visits at the four tertiary multidisciplinary clinics where the standards of care, including rehabilitation therapies, were consistent [30]. Individuals were measured in a single first assessment of passive bilateral lower-limb ROM, including hip extension, hip flexion, knee extension, knee flexion, ankle dorsiflexion and plantar flexion, as previously described [10]. All ROM measures were completed in supine position. Hip extension was measured using the Thomas Test, with the measurement taken before movement was felt on the contralateral anterior superior iliac spine. Hip flexion was measured with the opposite hip extended and the hips in neutral rotation. Stabilization was provided at the hips to prevent posterior pelvic tilt. Knee extension was measured with the hip extended, and knee flexion was measured with the hip flexed to 90 degrees. Both ankle plantar flexion and dorsiflexion were measured in the subtalar neutral position: plantar flexion with the knee extended with a bolster or towel roll under the calf to ensure the heel was off the table, and dorsiflexion with the knee flexed to 90 degrees [10].

### 2.4. Analysis

Assessments that were collected in positions where the ROM would be influenced by biarticular muscles were not included in the analysis. These included knee flexion in the prone position, knee extension with the hip flexed at 90 degrees and ankle dorsiflexion with the knee extended. Functional assessments completed during the same visit included the six-minute walk test (6 MWT), a test of ambulatory function [31], and the Hammersmith Functional Motor Scale—Expanded (HFMSE), a disease-specific scale used to evaluate gross motor function in SMA children and adults [32]. Data were collected from 2014 to 2019, and it was confirmed that all participants at the time of ROM measurement had not received disease-modifying therapy (DMT). ROM measures were recorded on both the left and right sides and, in line with a previous ROM study, the average was calculated and used for analysis [10]. The normal physiological values for each passive joint ROM were defined in the previous ROM study, and all measures above this ROM were considered hypermobile for this analysis [10,19]. The hypermobile ROM values were >10° hip extension and >130° hip flexion, >5° knee extension and >130° knee flexion, >20° ankle dorsiflexion and >50° plantarflexion. Alternate hypermobile cut points for children (0–9 years) and adolescents (10–19 years) were used to determine the frequency of hypermobile measures in these subgroups [33]. Outliers that were 2 standard deviations above the mean were queried with affiliated sites. Where possible, data entry errors were rectified, and if not, they were excluded. Spearman’s rank correlation coefficient was used to estimate the correlations between ROM measures that met the criterion for JH and the functional outcome measures (6 MWT or HFMSE), as the data were not normally distributed. Due to the small sample size and the multiple potentially confounding variables that were not assessed, further analyses by subgroup were not performed.

## 3. Results

A total of 143 participants with bilateral lower-limb passive ROM measures and at least one functional outcome measure (6 MWT or HFMSE) were included in the analysis. The clinical characteristics of the participants are included in Table 1. The majority were type II (50%) and type III (34%), as compared to type I participants (16%), and 23% (*n* = 33) were ambulant. There were 26 participants (18%) who had undergone spinal surgery for the management of scoliosis. Insufficient height and weight data were available to include body mass index (BMI) as a potential variable of interest.

**Figure 1:**
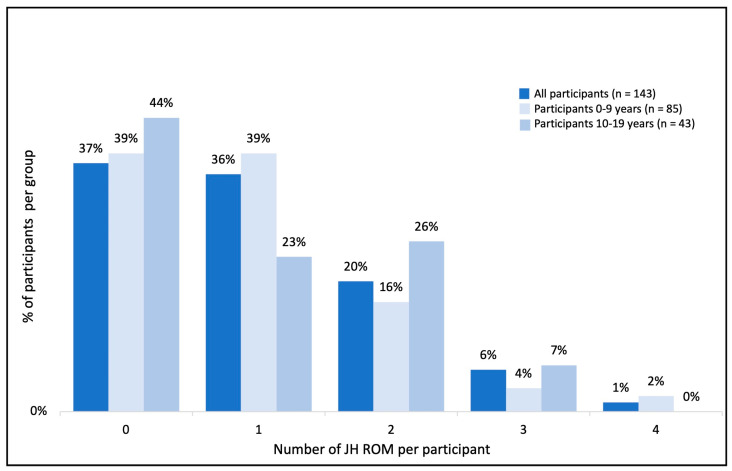
Frequency histogram of JH measures in the lower limb in the whole cohort and age subgroups: 0–9 years and 10–19 years. There were 6 measures in total, with a minimum of 0 measures of JH and a maximum of 5 measures classified as JH. JH, joint hypermobility.

Of the 143 participants, 123 (86%) had at least one ROM measure that was hypermobile, 72 (50%) had two or more, and 32 (22%) had three or more. The average age of those with three or more JH ROMs was 8.1 years (range 1.1–45.2), and 47% were type II and 53% were type III. Using age-adjusted JH cut points, the percentage of individuals with JH and the number of ROMs involved for the 0–9 year age group (*n* = 85), the 10–19 year age group (*n* = 43) and the whole cohort (*n* = 143) are presented in Figure 1. With higher JH cut points, the percentages of children with one (0–9 years = 39%; 10–19 years = 23%, all = 36%) or two joints (0–9 years = 16%; 10–19 years = 26%, all = 28%) with JH when compared to the whole cohort were similar.

Knee flexion was the most frequent measure that was hypermobile (*n* = 108, 79%), with a median age of 10.2 years (range 0.7–45.2) (Table 2). Ankle plantarflexion was the second most frequent measure (*n* = 71, 62%), with a median age of 8.3 years (range 1.1–26.2). For knee flexion JH and ankle plantarflexion JH, 49% of participants were type II, and 43% and 42%, respectively, were type III. In those who were classified as having knee flexion JH, the average ROM was 145 degrees (normal = 130 degrees), and for ankle plantarflexion, it was 63 degrees (normal = 50 degrees) (Table 2). JH prevalence presented in the pattern of knee > ankle > hip. 

**Figure 2:**
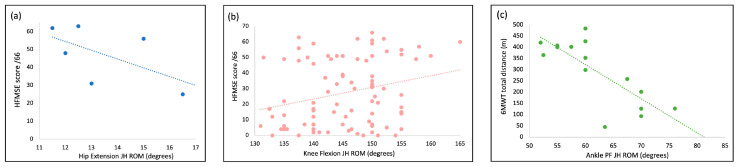
Associations between JH ROM and measures of function: (**a**) moderate inverse association between hip extension JH and HFMSE score (blue); (**b**) small positive association between knee flexion JH and HFMSE (pink); and (**c**) moderate inverse relationship between ankle PF and 6 MWT (green). JH, joint hypermobility; ROM, range of motion; HFMSE, Hammersmith Functional Motor Scale—Expanded; PF, plantar flexion; 6 MWT, six-minute walk test.

There was a moderate inverse association between the HFMSE score and hip extension JH (r = −0.60, *p* = 0.21; *n* = 6) (Figure 2a). A small positive association between the HFMSE score and knee flexion was observed (r = 0.24, *p* = 0.02, *n* = 89) (Figure 2b). There was a moderate inverse association between the 6 MWT distance and ankle plantar flexion JH (r = −0.73, *p* = 0.002; *n* = 15) (Figure 2c). No associations were detected between hip extension or knee flexion JH and the 6 MWT distance, nor between ankle plantarflexion JH and the HFMSE score. No associations were detected between hip flexion, knee extension or ankle dorsiflexion JH and either the HFMSE score or 6 MWT distance. 

## 4. Discussion

Joint hypermobility exists in SMA and is prevalent in the lower limbs, affecting 87% of individuals in at least one joint measure. Even when considering age, a known factor influencing JH, more than half of the children in this study had JH in one or more ROMs measured. Contractures have been sufficiently described in untreated SMA cohorts; however, this is the first study to identify excessive joint ROM beyond normal limits. While many individuals had one joint with hypermobility in the lower limbs, it was common in multiple joints, with 50% exhibiting two or more joints that were hypermobile and 22% with three or more measures of JH, indicating that this is not a unique occurrence. 

In addition to prevalence, JH in this sample was also associated with function. Knee flexion JH was the measure most frequently identified as hypermobile, and it was weakly associated with the HFMSE score. The presence of knee flexion JH might be explained by reduced leg muscle mass, resulting in an increased approximation of the tibia to the femur. However, this analysis was unable to include measures like leg circumference or BMI to determine whether this was a factor influencing knee flexion JH. The selective vulnerability of knee extensor muscles, resulting in an imbalance between the agonist and antagonist, could also contribute to knee flexion JH [17,34]. A future assessment of ROM that includes an assessment of strength may assist in identifying this as a potential contributing factor for JH in the knee as well as other joints where JH exists. 

At the ankles, plantar flexion JH was inversely associated with the 6 MWT distance. However, there was no significant relationship found between ankle plantar flexion JH and the HFMSE score, indicating that the influence of ankle plantar flexion JH on function in SMA may only be relevant to ambulant individuals. One possible explanation for the inverse association between ambulatory function and plantar flexion JH is increased fatigability. This is a known consequence affecting walking and jumping in individuals with generalized joint hypermobility [35] and is a frequent and clinically relevant problem in other hypermobile conditions like Ehlers–Danlos syndrome [17]. Fatigability could be attributed to the higher demands on active joint stabilization mechanisms that may be used as compensation during walking [35,36,37]. While further research needs to be carried out to understand this association, rehabilitation efforts focused on active strengthening may mitigate potential impacts on ambulatory function in SMA. 

There was a moderate inverse association between hip extension JH ROM and the HFMSE score, but this should be interpreted with caution due to the small number of individuals with hip extension JH. As with ankle plantar flexion, this association may suggest that JH at the hip requires more effort to stabilize the joint, and in individuals with muscle weakness, this may not be achievable, impacting function. Other factors, like the influence of joint subluxation or prior surgery, including scoliosis surgery, are worthy of investigation to understand their influence upon hip extension ROM and any associated impact on function. 

In this cross-sectional analysis, JH was most prevalent in the knees, followed by the ankles, and then the hips. The presentation of contractures follows this pattern in type II but presents differently in type III SMA (knee > ankle > hip; ankle > knee > hip, respectively) [11,12]. The combination of contractures and JH has been described in other neuromuscular disorders (NMDs), including Ullrich congenital muscular dystrophy and genetic disorders of connective tissue [17]. They have a similar distribution of JH, and the co-existence of dislocations and contractures is described, often affecting proximal joints [17]. These similar, yet genetically disparate, conditions provide a framework to which SMA and the role of SMN can be applied. The coexistence of JH and contractures in SMA has only been acknowledged in the upper limbs in one expert orthopedic perspective publication [38], and the role of SMN in various tissues, including the joint structure, is not well understood. The framework developed in other NMDs suggests that acknowledging the presence of JH may help to discriminate between conditions and direct future treatment [17,34].

Distinguishing between joint hypermobility and generalized joint hypermobility is an important factor, which this study does not explore. Standard GJH measurement using the Beighton score involves tests of both the upper and lower limbs and standing with legs straight and placing two palms flat on the floor, demonstrating the hypermobility of the axial skeleton [15]. This is not achievable for many individuals with SMA, and while there is acceptable reliability, the validity of the Beighton score and all other measures of GJH is not satisfactory [39]. Efforts continue to improve methods of evaluating GJH; however, goniometry for JH remains a gold standard measure of evaluation. 

### Limitations

This study is not without limitations. It is worth recognizing that some participants did not have all six lower-limb joint ROM measures or both measures of function, which, in some cases, reduced the number of cases available for statistical analysis. The frequency of multiple measures of JH (Figure 1) did not account for individuals who did not have all six measures; therefore, these numbers are conservative, and the prevalence may be higher. Age is an important factor in SMA and is known to influence JH. While the frequency of JH in the age-adjusted subgroups was reported in this analysis, age should ideally be included as a factor in future studies aimed at further understanding the impact of JH in SMA.

This cross-sectional analysis aimed to identify the presence of lower-limb JH in SMA, which may then be used to evaluate changes in the ROM over time. The trajectories of contractures, the normal ROM, or JH, as well as the impact of treatment, have not yet been evaluated. In the era of the availability of DMTs for SMA, it is important to first understand the natural history in order to subsequently evaluate the impact of treatments. There are multiple influences on a joint that impact the ROM that may be specific to flexion and extension. For example, decreased muscle mass of the calf and posterior thigh muscles may have resulted in a greater prevalence of knee flexion JH in this study. Whereas extensor ROM may be influenced by specific soft tissue extensibility, these factors were not evaluated in this study. Future studies should include assessments of the relative contributions of factors such as age, capsular and tissue extensibility, and biarticular muscles upon JH. Understanding the contribution of extension JH to the sagittal arc of the ROM may enhance clinical interpretation. The utilization of the ROM during various aspects of function, including gait, is an important consideration for this future work, particularly as more individuals remain ambulant. 

## 5. Conclusions

This analysis has shown that JH is prevalent in SMA and was seen in almost all participants in at least one lower-limb ROM. Many participants had multiple JH measures, indicating that it is not an isolated occurrence. The association of JH with both ambulatory and gross motor function varied based on the joint, indicating the complexity of the relationship. Future research should aim to better understand the influence of JH on function and build on these findings by describing the presence of a normal ROM, contractures, and JH in both the upper and lower limbs in individuals with SMA, as well as their trajectories over time. We can then aim to better understand the complexity of ROM changes over time, including the subsequent impact of DMT. This will direct future standard of care recommendations that improve the function of individuals with SMA.

## Figures and Tables

**Table 1:** Participant characteristics.

	Mean (Range)
**Age** (years)	9.8 (0.5–45.2)
	***n* (%)**
**Sex** Female Male	62 (43) 81 (57)
**Ambulatory Status** Ambulant Non-ambulant Missing	33 (23) 102 (71) 8 (6)
**SMA Type** 1 2 3	23 (16) 71 (50) 49 (34)
**Scoliosis Surgery** Yes No Missing	26 (18) 103 (72) 14 (10)

**Table 2:** Participant characteristics with joint hypermobility by range of motion assessment.

	Hip Extension > 10°	Hip Flexion > 130°	Knee Extension > 5°	Knee Flexion > 130°	Ankle DF > 20°	Ankle PF > 50°
Total assessments	134	135	135	137	134	114
**Hypermobile *n* (%)**	**7 (5)**	**26 (19)**	**2 (1)**	**108 (79)**	**19 (14)**	**71 (62)**
Age (years) median (range)	8.3 (1.2–25.0)	9.6 (1.3–45.2)	4.3 (3.1–5.5)	10.2 (0.7–45.2)	5.1 (0.7–45.2)	8.3 (1.1–26.2)
Sex *n* (%)						
female	2 (29)	14 (52)	1 (50)	44 (41)	11 (58)	33 (46)
male	5 (71)	13 (48)	1 (50)	64 (59)	8 (42)	38 (54)
SMN2 copy number *n* (%)						
1	0 (0)	1 (4)	0	2 (2)	0	1 (1)
2	1 (14)	5 (19)	0	8 (7)	3 (16)	8 (11)
3	3 (43)	6 (23)	0	38 (35)	7 (37)	28 (39)
>3	1 (14)	4 (15)	0	8 (7)	0	7 (10)
Unknown	2 (29)	10 (38)	2 (100)	52 (46)	9 (47)	27 (38)
SMA type *n* (%)						
1	1 (14)	2 (8)	0 (0)	9 (8)	2 (11)	6 (8)
2	3 (43)	10 (38)	1 (50)	53 (49)	12 (63)	35 (49)
3	3 (43)	14 (54)	1 (50)	46 (43)	5 (26)	30 (42)
Ambulatory status *n* (%)						
Non-ambulant	4 (57)	14 (54)	1 (50)	70 (65)	14 (67)	43 (61)
Ambulant	3 (43)	12 (46)	1 (50)	31 (29)	3 (14)	24 (34)
Missing				7 (6)	4 (19)	4 (6)
6 MWT *n* (mean, range)	1 (228m, 228)	5 (256m, 142–318)	1 (93m, 93)	20 (273m, 45–483)	2 (338m, 268–407)	15 (293m, 45–483)
HFMSE *n* (mean, range)	6 (48, 25–63)	23 (37, 2–62)	1 (51, 51)	89 (27, 0–66)	17 (25, 1–52)	81 (30, 0–66)

DF, dorsiflexion; PF, plantarflexion; n, number; SMN2, survival motor neuron 2; SMA, spinal muscular atrophy; 6MWT, six-minute walk test; m, meters; HFMSE, Hammersmith Functional Motor Scale-Expanded.

## Data Availability

The data presented in this study are available upon request from the corresponding author. The data are not publicly available due to privacy reasons.
